# Sunflowers and Malaria

**Published:** 1889-12

**Authors:** 


### SUNFLOWERS AND MALARIA.

In a recent issue of the Russian Medical journal, the *Meditzina*, a contributor draws attention to the common sunflower as an excellent and cheap substitute for quinine in the treatment of malarial fevers of all possible forms. The remedy has been from time immemmorial used for the purpose in the Russian, as well as Persian and Turkish popular medicines, and that mostly after the following plan: A flask is filled loosely with finely cut dry or recent flowers and stems, and then with vodka aqua vitæ. The hermetically corked vessel is left to stand under sun rays, or at some warm place, for two or three days. The tincture is then ready for use, and should be given as a small wineglassful three times a day. In recent cases, complete and permanent cure ensues in from one to three days; in most obstinate and inveterate cases, not later than a week. The remedy proved successful, even in such cases where quinine and other anti-malarial means failed.

